# Willingness to use nonpharmacologic treatments for musculoskeletal pain in the emergency department: a cross-sectional study

**DOI:** 10.1097/PR9.0000000000001027

**Published:** 2022-08-17

**Authors:** Stephanie A. Eucker, Shawna Foley, Sarah Peskoe, Alexander Gordee, Thomas Risoli, Frances Morales, Steven Z. George

**Affiliations:** a Department of Emergency Medicine and Department of Orthopaedic Surgery, Duke School of Medicine, Durham, NC, USA; b Emergency Department, University of Tennessee Medical Center, Knoxville, TN, USA; c Department of Biostatistics and Bioinformatics, Duke University, Durham, NC, USA; d University of Virginia School of Medicine, Charlottesville, VA, USA; e Department of Orthopaedic Surgery and Duke Clinical Research Institute, Duke University, Durham, NC, USA

**Keywords:** Musculoskeletal pain, Emergency medicine, Nonpharmacologic treatment, Patient-centered outcomes

## Abstract

**Objectives:**

Pain is an individual experience that should incorporate patient-centered care. This study seeks to incorporate patient perspectives toward expanding nonpharmacologic treatment options for pain from the emergency department (ED).

**Methods:**

In this cross-sectional study of adult patients in ED with musculoskeletal neck, back, or extremity pain, patient-reported outcomes were collected including willingness to try and prior use of various nonpharmacologic pain treatments, sociodemographics, clinical characteristics, functional outcomes, psychological distress, and nonmusculoskeletal symptoms. Least absolute shrinkage and selection operator regression identified variables associated with (1) willingness to try and (2) having previously tried nonpharmacologic treatments.

**Results:**

Responses were analyzed from 206 adults, with a mean age of 45.4 (SD 16.4) years. The majority (90.3%) of patients in ED were willing to try at least one form of nonpharmacologic pain treatment, with 70.4%, 81.6%, and 70.9% willing to try respective subcategories of active (eg, exercise), passive (eg, heat), and psychosocial (eg, prayer) modalities. Only 56.3% of patients had previously tried any, with 35.0%, 52.4%, and 41.3% having tried active, passive, and psychosocial modalities, respectively. Patient-level factors associated with willingness included pain in upper back, more severe pain-related symptoms, and functional impairments. The factor most consistently associated with treatment use was health care provider encouragement to do so.

**Conclusions:**

Patients in ED report high willingness to try nonpharmacologic treatments for pain. Higher pain severity and interference may indicate greater willingness, while health care provider encouragement correlated with treatment use. These findings may inform future strategies to increase the introduction of nonpharmacologic treatments from the ED.

## Abstract 2

Supplemental Digital Content is Available in the Text.

Patients in emergency department with musculoskeletal pain report high willingness to try nonpharmacologic treatments, and health care provider encouragement correlated with greater nonpharmacologic treatment use.

## 1. Introduction

Musculoskeletal pain accounts for a significant percentage of emergency department (ED) visits and is frequently characterized by high levels of pain intensity and functional impairments.^14,27,29^ Furthermore, poorly controlled musculoskeletal pain in the first 1 to 2 weeks after an ED visit has been associated with persistent pain and disability 3 months later.^10,12,14,19^ Despite numerous studies seeking to improve ED pain management through a variety of opioid and nonopioid medication strategies, most have demonstrated only modest improvements while in the ED with no clear evidence of the superiority or long-term efficacy of any specific class of medications.^2,4,7,14^ In particular, an analysis of 354 patients with low back pain enrolled in 2 robust randomized controlled trials comprising 4 different medication regimens demonstrated that despite treatment, 39% of patients across all arms reported continued functional impairments at 3 months.^10,11,14,19^ Furthermore, risk factors for persistent pain include higher levels of anxiety and psychological distress, greater impairment in ambulation, and traumatic etiology of pain, which may be better addressed using nonpharmacologic treatments, such as physical therapy and cognitive behavioral therapies.^35^ Numerous nonpharmacologic interventions have been shown to improve both short-term and long-term pain and functional outcomes in recent evidence syntheses and are now endorsed by clinical practice guidelines from a number of different national organizations for both acute and chronic pain.^4,14,22,25,31^ Additionally, a number of “proof of concept” studies indicate that nonpharmacologic pain treatments may be viable strategies for improving outcomes in patients in ED with both acute and chronic pain.^32^ However, few studies have evaluated the patient perspective toward initiating nonpharmacologic pain treatments from the ED.

Pain is an individualized biopsychosocial experience that should be treated using patient-centered management models.^1,17,28^ The range of nonpharmacologic options is broad and encompasses treatments that target different biological, psychological, and social domains. While these treatments are appealing options for pain management, the investigation and incorporation of nonpharmacologic strategies in the ED remains limited.^32^ A primary barrier to increasing the uptake of nonpharmacologic treatments in the ED is determining whether patients are willing to employ nonpharmacologic treatments in this setting. Therefore, it is important to identify and characterize willingness to try different nonpharmacologic treatment modalities to manage their musculoskeletal pain during or after their ED visit from the patient perspective.

The goals of this study are to (1) describe both willingness to try and having previously tried nonpharmacologic treatments in a cohort of patient presenting to an academic urban ED for musculoskeletal pain and (2) identify demographic, clinical, psychosocial, and pain characteristics associated with willingness to try or previous use of nonpharmacologic treatments for pain. This novel information will provide foundational data that could be used to incorporate the patient's perspective into effectively initiating nonpharmacologic treatments from the ED.

## 2. Methods

### 2.1. Study design, setting, and selection of participants

This was an analysis of cross-sectional data on pain management expectations of patients presenting to an academic urban ED for undifferentiated musculoskeletal pain.^13^ We collected patient-reported outcome (PRO) data from June 2018 to October 2019 on a convenience sample of adult patients in ED (18 years or older) with triage levels 3 to 5 who presented with a chief complaint of neck, back, or extremity pain deemed to be musculoskeletal (ie, not due to an alternative etiology such as infection, deep vein thrombosis, ischemia, and the like) by the treating ED provider (attending, resident, or physician assistant). Patients were excluded if they were non-English speaking or not able to consent. Recruitment occurred between 9 am and 9 pm, Monday to Friday, and occasionally on weekends for patients in all ED care areas and in the waiting room. The study was approved by the University Health System Institutional Review Board and follows the STROBE reporting guidelines.

### 2.2. Study protocol and measures

Patients who met inclusion criteria were approached by a research associate during their ED visit after their initial assessment by an ED provider. Patients answered a series of questions in a 25- to 35-minute online questionnaire delivered using tablet. As few PRO measures have been validated specifically in patients in ED presenting for pain, tools previously validated in the other settings most closely approximating ED (ie, clinics seeing patients with acute pain) were used for this study as described below.

#### 2.2.1. Willingness to try nonpharmacologic treatments

We collected information about nonpharmacologic treatments including (1) what treatments patients were willing to try and (2) which treatment they had tried for their pain (Appendix 1, available as supplemental digital content at http://links.lww.com/PR9/A165). Patient responses for treatments they were willing to try and had tried were then grouped into 3 categories used in a systematic review of this topic^32^:

(1) Active methods: exercise, physical therapy, walking, yoga
(2) Passive methods: application of cold or heat, acupuncture, acupressure, massage
(3) Psychosocial methods: deep breathing, distraction, imagery, meditation, mindfulness, music, prayer, relaxation, support groups.

#### 2.2.2. Patient-reported outcomes

(1) Patient demographics, including age, sex, race, ethnicity, employment, marital status, education, income, and insurance status.
(2) Pain characteristics including anatomical location of pain, duration of current episode of musculoskeletal pain, and history of episodes of musculoskeletal pain. Severity of pain was captured using the Brief Pain Inventory, which includes the 0 to 10 point numerical rating scale of current pain commonly used in ED assessments, as well as worst, best, and average pain in the past 24 hours on the 0 to 10 scale.^20^
(3) Optimal Screening for Prediction of Referral and Outcome Review of Systems tool, an assessment tool for systemic symptoms shown to predict pain and quality-of-life outcomes after musculoskeletal care episodes in outpatient physical therapy settings.^15,16^
(4) Optimal Screening for Prediction of Referral and Outcome Yellow Flag tool, an assessment tool for measuring psychological response to pain including pain coping, vulnerability, and resilience. It has been shown to be reliable and predict pain and functional outcomes after musculoskeletal care in outpatient physical therapy settings.^5,16,24^ The Optimal Screening for Prediction of Referral and Outcome Yellow Flag 4-factor subscores were calculated as described in the study of Butera et al. and consist of Negative Mood, Pain Catastrophizing, Fear Avoidance, and Pain Acceptance/Self-efficacy.^5^
(5) PROMIS-29, a validated succinct assessment of patient-reported pain and functional outcomes in multiple domains including pain interference, sleep disturbance, physical function, and social function among others.^6,23^
(6) Musculoskeletal Outcomes Data Evaluation and Management System expectations subcomponent is a validated and reliable six-item questionnaire of patients' expected outcomes from treatment for their musculoskeletal disorders.^9,36^

### 2.3. Data analysis

Descriptive statistics were calculated to examine patient characteristics across the cohort. Distributions and frequencies for categorical measures are presented using counts and percentages for nonmissing data. Continuous measures are presented using means, standard deviations, medians, the 25th and 75th percentiles (interquartile range), and the range (min and max). Differences between patients who have tried at least one nonpharmacologic treatment were assessed using the Wilcoxon rank-sum test for continuous measures and the χ^2^ test for categorical measures. Proportional differences between those who were willing to try and those who have tried nonpharmacologic treatments were measured using McNemar test due to the paired nature of the groups (ie, patients could have been willing to try and have already tried the treatments).

Six separate logistic regression models were used to model both the willingness to try and having tried in the past at least one treatment in each of the 3 categories of methods: active, passive, and psychosocial. To identify characteristics that were most associated with willingness to try nonpharmacologic treatments, least absolute shrinkage and selection operator (LASSO) was implemented as a variable selection procedure.^34^ The tuning parameter yielding the lowest mean square error was chosen using a 10-fold cross-validation technique for each model. All possible covariates were considered for the LASSO procedure, and an indicator variable was included in the analysis for any covariate with missing observations. Unadjusted and adjusted effect estimates of the covariates chosen by the LASSO procedure were obtained from nonpenalized logistic regression models. Given the exploratory and hypothesis generating nature of this analysis, no inference on final selected models was performed. The data analysis for this article was generated using SAS/STAT software version 14.3 and SAS software version 9.4 for Windows (Copyright 2016 SAS Institute Inc. SAS and all other SAS Institute Inc. product or service names are registered trademarks or trademarks of SAS Institute, Inc, Cary, NC). We additionally used the R programming language version 4.0.2 in our analysis (R Core Team [2020]. R Foundation for Statistical Computing, Vienna, Austria).

## 3. Results

### 3.1. Characteristics of study subjects

**Figure 1.**
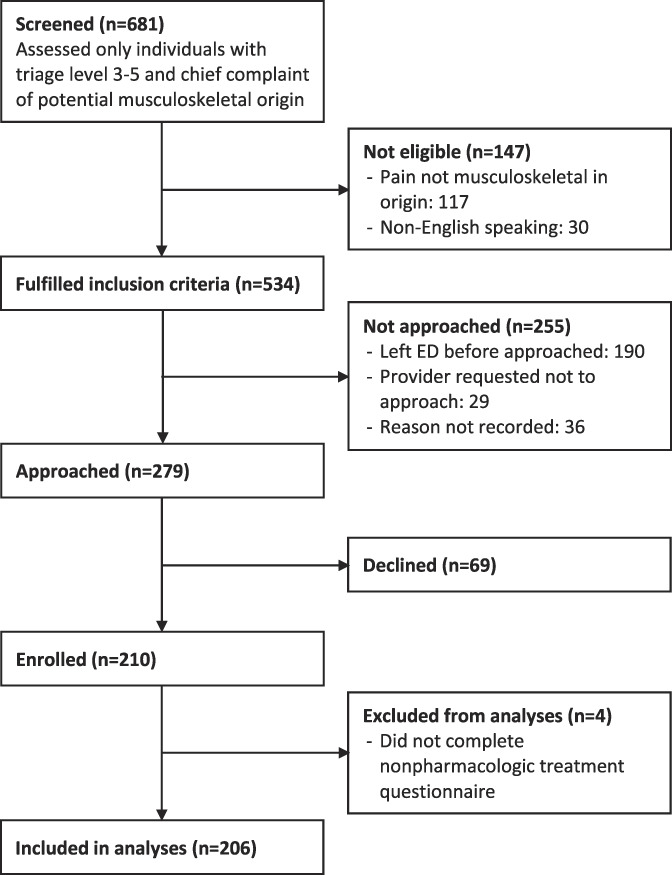
Flowchart depicting patient screening, enrollment, and inclusion in analyses.

Of a total of 681 screened patients, 279 fulfilled inclusion criteria and were able to be approached, and 210 patients enrolled (Figure 1). Of these, 206 patients provided complete responses and were included in the analysis. No additional patients were excluded from analysis due to missing data; rather, missingness was included as a variable for covariates in LASSO analysis. Patient characteristics are summarized for the total cohort, by willingness to try, and by previous use of any nonpharmacologic treatments for pain (Table 1).

**Table 1:** Patient characteristics.

Patient characteristics	Willing to try at least one nonpharmacologic treatment?		Have tried at least one nonpharmacologic treatment?		Total (N = 206)	*P* *
	No (N = 20)	Yes (N = 186)	No (N = 90)	Yes (N = 116)		
Age (in years)						
Mean (SD)	44.8 (16.5)	45.5 (16.5)	42.8 (17.4)	47.5 (15.4)	45.4 (16.4)	0.899^1^, 0.034^1^
Range	18–67	19–87	18–87	20–77	18–87	
Sex						
Female	5 (25.0%)	104 (55.9%)	41 (45.6%)	68 (58.6%)	109 (52.9%)	0.008^2^, 0.062^2^
Male	15 (75.0%)	82 (44.1%)	49 (54.4%)	48 (41.4%)	97 (47.1%)	
Racial group						0.598^2^, 0.731^2^
Black or African American	13 (68.4%)	100 (55.6%)	49 (57.0%)	64 (56.6%)	113 (56.8%)	
White or Caucasian	5 (26.3%)	69 (38.3%)	31 (36.0%)	43 (38.1%)	74 (37.2%)	
Asian, Native American, or Pacific Islander	0 (0%)	5 (2.9%)	2 (2.3%)	3 (2.7%)	5 (2.5%)	
More than one race	1 (5.3%)	6 (3.3%)	4 (4.7%)	3 (2.7%)	7 (3.5%)	
Missing	1 (5.0%)	6 (3.2%)	4 (4.4%)	3 (2.6%)	7 (3.4%)	
Ethnic group						0.584^2^, 0.121^2^
Hispanic or Latino	2 (10.5%)	12 (7.1%)	9 (10.7%)	5 (4.8%)	14 (7.4%)	
Missing	1 (5.0%)	16 (8.6%)	6 (6.7%)	11 (9.5%)	17 (8.3%)	
Current employment status						0.028^2^, 0.518^2^
Full-time employed	3 (15.8%)	81 (44.8%)	38 (43.2%)	46 (41.1%)	84 (42.0%)	
Part-time employed	4 (21.1%)	26 (14.4%)	15 (17.0%)	15 (13.4%)	30 (15.0%)	
Unemployed	10 (52.6%)	45 (24.9%)	25 (28.4%)	30 (26.8%)	55 (27.5%)	
Retired	2 (10.5%)	29 (16.0%)	10 (11.4%)	21 (18.8%)	31 (15.5%)	
Missing	1 (5.0%)	5 (2.7%)	2 (2.2%)	4 (3.4%)	6 (2.9%)	
Level of education completed						0.079^2^, 0.209^2^
Less than high school	4 (22.2%)	17 (9.3%)	10 (11.4%)	11 (9.8%)	21 (10.5%)	
Graduated from high school	7 (38.9%)	58 (31.9%)	34 (38.6%)	31 (27.7%)	65 (32.5%)	
Some college	6 (33.3%)	50 (27.5%)	26 (29.5%)	30 (26.8%)	56 (28.0%)	
Graduated from college or more	1 (5.6%)	57 (31.3%)	18 (20.4%)	40 (35.7%)	58 (29.0%)	
Missing	2 (10.0%)	4 (2.2%)	2 (2.2%)	4 (3.4%)	6 (2.9%)	
Approximate household income						0.177^2^, 0.435^2^
Less than $20,0000	10 (58.8%)	53 (31.9%)	32 (40.5%)	31 (29.8%)	63 (34.4%)	
$20,000–$35,000	4 (23.5%)	40 (24.1%)	16 (20.3%)	28 (26.9%)	44 (24.0%)	
$35,001–$50,000	2 (11.8%)	29 (17.5%)	15 (19.0%)	16 (15.4%)	31 (16.9%)	
$50,001–$70,000	0 (0.0%)	13 (7.8%)	5 (6.3%)	8 (7.7%)	13 (7.1%)	
Greater than $70,000	1 (5.9%)	31 (18.7%)	11 (13.9%)	21 (20.2%)	32 (17.5%)	
Missing	3 (15.0%)	20 (10.8%)	11 (12.2%)	12 (10.3%)	23 (11.2%)	
Location of primary current pain						0.606^2^, 0.036^2^
Neck	2 (11.1%)	17 (9.1%)	13 (14.8%)	6 (5.2%)	19 (9.3%)	
Upper back	0 (0.0%)	16 (8.6%)	4 (4.5%)	12 (10.3%)	16 (7.8%)	
Lower back	7 (38.9%)	53 (28.5%)	23 (26.1%)	37 (31.9%)	60 (29.4%)	
Arm	3 (16.7%)	23 (12.4%)	15 (17.0%)	11 (9.5%)	26 (12.7%)	
Leg	6 (33.3%)	77 (41.4%)	33 (37.5%)	50 (43.1%)	83 (40.7%)	
Missing	2 (10.0%)	0 (0.0%)	2 (2.2%)	0 (0.0%)	2 (1.0%)	
Duration of current pain (# of days)						0.048^1^, 0.010^1^
Median (IQR)	7.5 (2, 30)	3 (1, 10)	2 (1, 5)	4 (1, 24)	3 (1, 12)	
Missing	2 (10.0%)	0 (0.0%)	2 (2.2%)	0 (0.0%)	2 (1.0%)	
Medication taken for current pain						0.384^2^, 0.105^2^
Yes	13 (81.3%)	130 (71.0%)	56 (65.9%)	87 (76.3%)	143 (71.9%)	
Missing	4 (20.0%)	3 (1.6%)	5 (5.6%)	2 (1.7%)	7 (3.4%)	
PROMIS: pain intensity						
Median (IQR)	7 (2, 8)	7 (5, 9)	6 (3, 9)	8 (6, 10)	7 (5, 9)	0.404^1^, 0.003^1^
Missing	9 (45.0%)	15 (8.1%)	14 (15.6%)	10 (8.6%)	24 (11.7%)	
PROMIS: pain interference						
Median (IQR)	59.7 (41.6, 70.4)	65.2 (55.7, 71.3)	58.7 (50.9, 67.6)	66.7 (59.9, 75.6)	65.1 (55.7, 71.3)	0.347^1^, 0.001^1^
Missing	9 (45.0%)	15 (8.1%)	14 (15.6%)	10 (8.6%)	24 (11.7%)	
PROMIS: physical function						
Median (IQR)	36.4 (27.2, 47.9)	32.2 (27.7, 38.6)	34.4 (27.9, 40.3)	32.2 (27.2, 37.4)	32.3 (27.5, 38.9)	0.150^1^, 0.093^1^
Missing	9 (45.0%)	13 (7.0%)	13 (14.4%)	9 (7.8%)	22 (10.7%)	

* *P* values for each characteristic indicate comparisons between respondents reporting yes vs no for willingness to try (top) and have previously tried (bottom) any nonpharmacologic treatment based on ^1^Wilcoxon, ^2^χ^2^, or ^3^equal variance *t* test.

IQR, interquartile range.

### 3.2. Willingness to try nonpharmacologic treatments for pain

Characteristics of those more willing to try any nonpharmacologic treatments included being female and having full-time employment (Table 1). Table 2 reports the number and percentage of patients reporting “willingness” and “have tried” for each of the specific subcategories of nonpharmacologic treatments. The overwhelming majority of patients (N = 186; 90.3%) were willing to try at least one form of nonpharmacologic pain treatment. The methods that patients mostly frequently reported willingness to try were cold packs (N = 127; 61.7%), heat (N = 123; 59.7%), and massage (N = 108; 52.4%) within the passive subcategory; physical therapy (N = 122; 59.2%) and exercise (N = 82; 39.8%) within the active subcategory; and prayer (N = 95; 46.1%), relaxation (N = 94; 45.6%), and deep breathing (N = 83; 40.3%) within the psychosocial subcategory.

**Table 2:** Nonpharmacologic treatments participants are willing to try by subcategory.

Nonpharmacologic treatment	Willing to try, N (%)	Have tried, N (%)	*P* *
Active			
Physical therapy	122 (59.2%)	45 (21.8%)	<0.001
Exercise	82 (39.8%)	41 (19.9%)	<0.001
Walking	76 (36.9%)	41 (19.9%)	<0.001
Yoga	40 (19.4%)	14 (6.8%)	<0.001
Any active	145 (70.4%)	72 (35.0%)	<0.001
Passive			
Cold pack	127 (61.7%)	75 (36.4%)	<0.001
Heat	123 (59.7%)	75 (36.4%)	<0.001
Massage	108 (52.4%)	48 (23.3%)	<0.001
Acupuncture	56 (27.2%)	10 (4.9%)	<0.001
Acupressure	39 (18.9%)	6 (2.9%)	<0.001
Any passive	168 (81.6%)	108 (52.4%)	<0.001
Psychosocial			
Prayer	95 (46.1%)	52 (25.2%)	<0.001
Relaxation	94 (45.6%)	48 (23.3%)	<0.001
Deep breathing	83 (40.3%)	43 (20.9%)	<0.001
Distraction	75 (36.4%)	42 (20.4%)	<0.001
Listen to music	73 (35.4%)	33 (16.0%)	<0.001
Meditation	58 (28.2%)	27 (13.1%)	<0.001
Imagery	44 (21.4%)	18 (8.7%)	<0.001
Mindfulness	40 (19.4%)	14 (6.8%)	<0.001
Support group	36 (17.5%)	6 (2.9%)	<0.001
Any psychosocial	146 (70.9%)	85 (41.3%)	<0.001
Any nonpharmacologic	186 (90.3%)	116 (56.3%)	<0.0001

* McNemar test.

Using LASSO regression analysis, we identified patient demographic, clinical, pain, and psychological factors that were most associated with willingness to try one or more nonpharmacologic methods of pain control within each of the 3 subcategories (Table 3). Patients willing to try active treatments (eg, physical therapy) were more likely to have pain in the upper back (adjusted odds ratio [aOR] 5.47) or in multiple regions (aOR 2.26), report pain and activity limitations every day for 3 months or more (aOR 2.17), and expect treatment to improve their ability to do everyday activities (aOR 1.46).

**Table 3:** Univariable and multivariable logistic regression of willingness to try nonpharmacologic treatments in each subcategory.

Patient characteristics	Active		Passive		Psychosocial	
	Univariable odds ratio	Multivariable odds ratio	Univariable odds ratio	Multivariable odds ratio	Univariable odds ratio	Multivariable odds ratio
Sex						
Female						
Male			*0.291*	*0.282*	*0.259*	*0.390*
Current employment status*						
Full-time employed						
Part-time employed						
Unemployed			*0.416*	*0.342*		
Retired						
Current marital status*						
Single						
Married			**3.041**	**2.377**		
Living with significant other						
Divorced/separated						
Widowed/widower						
Level of education completed*						
Less than high school						
Graduated from high school	*0.495*	*0.522*	*0.557*	*0.548*		
Some college					**1.327**	**2.408**
Graduated from college			**6.826**	**4.329**		
Some postgraduate course work or completed postgraduate degree						
Type of insurance						
Private						
Medicare					**2.273**	**3.637**
Medicaid						
Uninsured					*0.451*	*0.458*
Other (includes missing)					*0.260*	*0.264*
Location of primary current painful symptoms*						
Neck						
Upper back	**6.923**	**5.466**			**>999.999**	**>999.999**
Lower back					*0.516*	*0.388*
Arm						
Leg			**2.537**	**1.887**		
Experiencing pain symptoms anywhere else*						
Yes	**2.312**	**2.264**			**2.006**	**2.792**
No						
Onset of current painful symptoms*						
Gradual						
Sudden						
Traumatic			**1.699**	**2.757**		
Painful symptoms are work related*						
Yes					*0.422*	*0.736*
No						
Have you experienced ANY pain and activity limitations every day for the past 3 months?*						
Yes	**2.422**	**2.172**				
No						
Previous episodes of painful symptoms over the past year*						
Yes			**1.875**	**2.833**		
No						
Visited any other health care providers for current painful symptoms in the past year*						
Yes					**2.273**	**1.376**
No						
Missing	*<0.001*	*<0.001*	*<0.001*	*<0.001*	*<0.001*	*<0.001*
Body mass index			*0.976*	*0.974*		
What is the main reason you came to the emergency department today?*						
Want pain relief						
Want to know cause of pain						
No primary care available	**>999.999**	**>999.999**				
Other						
Treatment goals: relief from symptoms (1–5 Likert scale)			**1.284**	**1.383**		
Treatment goals: to do more everyday household or yard activities (1–5 Likert scale)	**1.406**	**1.464**				
PROMIS: pain interference					**1.039**	**1.035**
PROMIS: physical function			*0.952*	*0.950*		
PROMIS: fatigue					**1.040**	**1.032**
OSPRO yellow flag tool: 4-factor positive coping					**1.050**	**1.122**

Table 3 shows the unadjusted (univariable) and adjusted (multivariable) odds ratios (OR) found to be significant (*P* < 0.05) for each of the patient variables in each model corresponding to willingness to try any in the active, passive, or psychosocial subcategories of nonpharmacologic treatments. For instance, we see that in an unadjusted model, the OR of willingness to try any psychosocial treatment when comparing individuals who had painful symptoms in more than one body region was 2.006, but this OR increases to 2.792 once we adjust for all other variables selected in the final model.

Within each subcategory, only the characteristics chosen during the LASSO procedure (*P* < 0.05) were used in the regression models. Bold values indicate OR>1 and italics indicate OR<1, where the 95% confidence intervals did not cross 1 for any of these reported values. OR are not reported for nonsignificant variables (95% confidence interval crosses 1). For continuous variables, for example, body mass index, the odds ratio corresponds to the OR per one-point increase in the variable value.

* For these categorical variables, the missing category was not selected by LASSO and was combined with the other categories to make the reference group.

LASSO, least absolute shrinkage and selection operator.

Patients willing to try passive treatments (eg, acupuncture, massage therapy) were more likely to be female (aOR 3.55), married (aOR 2.38), not unemployed, report prior pain episodes (aOR 2.83), pain due to trauma (aOR 2.76), and expect treatment to provide relief from symptoms. Patients willing to try psychosocial treatments (eg, prayer, relaxation) were more likely to be female (aOR 2.56), have pain primarily in the upper back or in multiple regions (aOR 2.79) but less likely to have primarily low back pain (aOR 0.388), and report higher severity of fatigue and pain interference but also report higher pain acceptance and self-efficacy. Importantly, there were no differences by age, race, ethnicity, or income in reported willingness to try any of the categories of nonpharmacologic treatments.

### 3.3. Previous use of nonpharmacologic treatments for pain

Only 116 patients (56.3%) had actually tried a nonpharmacologic treatment for musculoskeletal pain (Table 2). Interestingly, the methods with the highest number of patients willing to try them were also the ones more people had previously tried. However, the number of people who had previously tried these methods was only half the number of those willing to try them: cold packs (N = 75; 36.4%), heat (N = 75; 36.4%), massage (N = 48; 23.3%), physical therapy (N = 45; 21.8%), prayer (N = 52; 25.2%), and relaxation (N = 48; 23.3%). Characteristics of those who had tried any nonpharmacologic treatments included older age, longer duration of symptoms, greater severity of pain, or activity limitations (Table 1). Those who had tried nonpharmacologic treatments were also more likely to have received encouragement from a nurse or doctor to do so and had a high rate of willingness (N = 113; 97.4%) to try at least one nonpharmacologic treatment.

Least absolute shrinkage and selection operator also identified patient factors most associated with previous use of a nonpharmacologic treatment within each of the 3 subcategories (Table 4). The most consistent overall factor associated with having tried any of the nonpharmacologic modalities was any encouragement by a doctor or nurse to do so (aOR range 2.98–4.22 for “sometimes” and aOR range 1.78–6.70 for “often” across all treatment subcategories). In addition, pain in the upper back, higher pain intensity, and greater pain interference with activities correlated with having tried treatments in each of the subcategories.

**Table 4:** Univariable and multivariable logistic regression of having tried nonpharmacologic treatments in each subcategory.

Patient characteristics	Active		Passive		Psychosocial	
	Univariable odds ratio	Multivariable odds ratio	Univariable odds ratio	Multivariable odds ratio	Univariable odds ratio	Multivariable odds ratio
Age (in y)	**1.029**	**1.021**			**1.025**	**1.015**
Sex						
Female						
Male			*0.539*	*0.869*	*0.520*	*0.678*
Current marital status						
Single						
Married						
Living with significant other			**6.204**	**10.388**	**4.413**	**9.083**
Divorced/separated			**2.217**	**2.344**	**4.707**	**6.708**
Widowed/widower						
Missing	**>999.999**	**>999.99**				
Level of education completed*						
Less than high school						
Graduated from high school			*0.577*	*0.901*		
Some college						
Graduated from college						
Some postgraduate course work or completed postgraduate degree	**3.263**	**2.494**				
Approximate household income*						
Less than $20,000						
$20,000 to $35,000						
$35,001 to $50,000						
$50,001 to $70,000						
Greater than $70,000			**1.909**	**2.310**	**2.053**	**2.529**
Type of insurance						
Private			**1.315**	**1.636**		
Medicare						
Medicaid						
Uninsured						
Other (includes missing)			*0.569*	*0.680*		
Location of primary current pain*						
Neck						
Upper back	**2.591**	**2.608**	**2.937**	**3.252**	**3.449**	**5.166**
Lower back						
Arm						
Leg						
Experiencing pain symptoms anywhere else*						
Yes	**2.056**	**1.692**				
No						
Duration of current pain (# of days)	**1.001**	***1.000***				
Average pain over past 7 days			**1.176**	**1.122**		
Painful symptoms due to a motor vehicle crash*						
No						
Yes			*0.457*	*0.481*		
**Have you experienced ANY pain and activity limitations every day for the past 3 months?** *						
Yes	**3.188**	**1.662**	**2.104**	**1.257**	**2.787**	**1.825**
No						
Previous episodes of painful symptoms over the past year*						
Yes			**1.995**	**1.178**	**2.221**	**1.148**
No						
Have taken medication for current pain						
Yes			**2.103**	**1.297**		
No						
Missing	*<0.001*	*<0.001*				
Functional comorbidity index (FCI)	**1.273**	**1.046**				
Visited any other health care providers for current painful symptoms in the past year*						
Yes			**2.445**	*0.898*	**2.829**	**1.215**
No						
What is the main reason you came to the emergency department today?						
Want pain relief						
Want to know cause of pain						
No primary care available			*0.400*	*0.307*		
Other	*<0.001*	*<0.001*				
Missing	*<0.001*	*<0.001*	*<0.001*	*<0.001*	*<0.001*	*<0.001*
How often did a nurse or doctor encourage you to use non-medicine methods?						
Missing					*0.316*	*0.409*
Never						
Sometimes	**4.051**	**4.223**	**3.067**	**3.390**	**2.765**	**2.984**
Often	**7.945**	**6.699**	**4.025**	**2.763**	**2.723**	**1.779**
Treatment expectations: relief from symptoms (1–5 Likert scale)	*0.69*	*0.768*	*0.695*	*0.885*	*0.683*	*0.679*
Treatment expectations: to do more everyday household or yard activities (1–5 Likert scale)			*0.727*	*0.665*		
PROMIS: pain intensity	**1.173**	**1.175**	**1.164**	**1.091**	**1.158**	**1.130**
PROMIS: pain interference	**1.052**	**1.012**	**1.050**	**1.003**	**1.063**	**1.031**
PROMIS: sleep			**1.040**	**1.008**	**1.045**	
PROMIS: fatigue			**1.035**	**1.013**	**1.044**	**1.005**
OSPRO red flag tool total score			**1.214**	**1.103**	**1.243**	**1.002**

Table 4 shows the unadjusted (univariable) and adjusted (multivariable) odds ratios (OR) found to be significant (*P* < 0.05) for each of the patient characteristics and measures in each model corresponding to having previously tried any in the active, passive, or psychosocial subcategories of nonpharmacologic treatments. For instance, we see that in an unadjusted model, the OR of willingness to try any active treatment when comparing individuals who had painful symptoms in more than one body region was 2.056, but this OR decreases to 1.692 once we adjust for all other variables selected in the final model.

Within each subcategory, only the characteristics chosen during the LASSO procedure (*P* < 0.05) were used in the regression models. Bold values indicate OR>1 and italics indicate OR<1, where the 95% confidence intervals did not cross 1 for any of these reported values. OR are not reported for nonsignificant variables (95% confidence interval crosses 1). For continuous variables, for example, age (in y), the odds ratio corresponds to the OR per one-point increase in the variable value.

* For these categorical variables, the missing category was not selected by LASSO and was combined with the other categories to make the reference group.

LASSO, least absolute shrinkage and selection operator.

## 4. Discussion

Our study provides novel information for ED providers who want to incorporate the patient perspective for offering nonpharmacologic pain treatments in alignment with clinical practice guideline recommendations for musculoskeletal pain management.^22,25,30,31^ A primary finding of this cross-sectional study is that more than 90% of patients seeking care in the ED reported a willingness to try at least one nonpharmacologic treatment. In contrast, only 56% of patients reported having tried at least one nonpharmacologic treatment. The gap between what patients are willing to try for pain management and what they have been exposed to suggests opportunities for increasing uptake of nonpharmacologic treatments. In particular, our data show that patients who received encouragement by a health care provider were much more likely to use nonpharmacologic treatments. These data establish that there is strong interest in nonpharmacologic treatments for patients seeking care in the ED. It was beyond the intended scope of this study to determine which treatments would be feasible to deliver in the ED vs outside the ED; however, that topic remains an important avenue for future research.

These findings coupled with clinical guidelines recommending the use of nonpharmacologic treatments for both acute and chronic pain lend support to health care system efforts to reduce opioid prescribing by initiating nonpharmacologic treatments in the ED setting.^22,25,30,31^ In addition, these findings potentially have relevance for different stakeholder groups involved in the management of musculoskeletal pain. For providers, these results indicate that each patient will have a unique receptiveness to various types of nonpharmacologic modalities in managing their pain. For health systems, they demonstrate the need to implement structured ways to deliver nonpharmacologic care in the ED. For researchers, they highlight the need to further develop the evidence base to determine (1) which nonpharmacologic treatments improve meaningful outcomes; (2) which are cost effective; and (3) which can be feasibly accessed either in an ED setting by either (a) co-location with other provider types and/or (b) through a streamlined rapid referral process. For patients, these findings identify nonpharmacologic treatments that could be further explored for their feasibility to be delivered either in the ED or by a structured referral process. Some treatments endorsed by the patients (eg, prayer) may not be feasible for many settings, but they were mentioned as part of the patient perspective, so should be given future consideration when designing innovative pain management care pathways that start in the ED. Finally, these findings underscore the need for future development of prediction tools to identify which patients in ED will go on to develop persistent pain and benefit most from early intervention, as well as concise shared decision-making models that can be implemented by ED providers to better direct subsequent individualized treatment options for pain management.

Active treatment modalities, including physical therapy, exercise, and yoga, are supported by a growing body of evidence as effective pain treatments in non-ED settings and show high potential for benefit in patients in ED.^8,18,21^ Our data suggest that patients would be open to ED recommendations and referrals to physical therapy, structured exercise programs, yoga, and other similar strategies that align with the most recent ambulatory care guidelines. There was an overall high (70.4%) willingness to try at least one of these active modalities regardless of age, sex, race, or socioeconomic factors; however, only 35.0% reported previous use of any of these methods. Additionally, those with a higher pain and activity limitation burden who may benefit most from these interventions were more willing to try them. Furthermore, those seeking care for musculoskeletal pain due to lack of primary care provider have greater willingness and could be particularly well-served by ED initiatives to connect them to these treatments.

Patients in ED also report high (81.6%) willingness to use passive treatment methods including application of cold or heat, massage, and acupuncture. Patients are familiar with these methods, with 52.4% having previously tried at least one of these treatments, in particular cold and heat, which are easily adopted in the ED or at home with minimal cost and without need for prescription. Many of these treatment strategies show moderate efficacy and are recommended in recent practice guidelines, as they are feasible and economical adjuncts for short-term pain relief and reductions in opioid use.^8,28,32^ Our results further support these guidelines by providing evidence that ED patients who may most benefit from these interventions (eg, traumatic leg injuries such as strains and sprains) are also more willing to use them.

Psychosocial treatments for musculoskeletal pain that were advocated for by patients included prayer, relaxation, deep breathing, distraction, music, and meditation. These treatments are becoming more widely accepted, and increasing evidence supports their efficacy as pain management options.^8,33^ Furthermore, many of these treatments can be performed at home without the burden of additional health care visits. Our data show that patients in ED also report high (70.9%) willingness to try these treatments, although only 41.3% had previously tried any of them. Specifically, prayer and relaxation were identified as the 2 most common methods patients were willing to try in this subcategory. In addition, the high rate of willingness to try active modalities suggests that many individuals would be open to either or both of these approaches. In particular, yoga, group exercise, and physical therapy when combined with cognitive behavioral approaches,^3^ offer a combination of active and psychosocial therapies.^26^ Finally, those patients experiencing greater fatigue or interference with activities due to pain were more willing to try psychosocial treatments. This is perhaps because they may be less physically able to engage in active treatments, and thus they may derive particular benefit from psychosocial methods.

The strengths of this study include a demographically diverse patient population in ED, which is reflective of our general population in ED regarding age, sex, race, ethnicity and insurance status, the use of previously validated PRO instruments to measure multiple biopsychosocial variables of interest, and the use of LASSO regression modeling as a robust tool for identifying patient-level factors associated with willingness to try nonpharmacologic pain treatment modalities. While a large number of variables were selected by the LASSO and should be considered as potential predictors of willingness and having tried nonpharmacologic pain treatment modalities, the results are informative for hypothesis generation for future inferential studies.

The limitations of this study included a limited list of predetermined nonpharmacologic modalities due to the additional time and cognitive burden that an exhaustive list might impose on participants. Patients may have been unfamiliar with some of the terms, which may have limited the number of respondents willing to try them. Another limitation is that some terms were generic or linked to a specific provider type (eg, physical therapy) that could deliver a variety of nonpharmacologic treatments. This lack of specificity could have caused confusion for those wanting more detail on the type or aspect of physical therapy they would receive. In addition, this specific set of questions (Appendix 1, available as supplemental digital content at http://links.lww.com/PR9/A165) has not been previously validated in the ED setting, and other treatment modalities were able to be entered as free text (Appendix 2, available as supplemental digital content at http://links.lww.com/PR9/A165). The treatments that patients reported they would be willing to try may not match treatments they receive when seeking care. The selection of PRO instruments and variables measuring clinical and biopsychosocial factors was necessarily limited to prevent overburdening participants. This was a cross-sectional study design that offered insights into the patient perspective only at the time of their ED visit. We did not follow patients to determine their likelihood of pursuing any of these pain management options after discharge from the ED. Furthermore, and consistent with all cross-sectional studies and exploratory analysis, the associations reported between variables do not infer causality. Additionally, treatments identified by patients as being willing to try would also need to be tested in randomized trials to determine whether they are efficacious. Finally, this study was conducted at a single center with a relatively small sample size in a convenience sample during limited hours, so the results might not generalize to other settings or across specific demographic characteristics. However, our recruitment encompassed peak ED arrival times and reflected our general ED demographics, suggesting that this potential bias was limited. While the rates of acceptance of individual nonpharmacologic interventions may be region or population specific, the large percentage of acceptance of at least one item within each major subcategory of physical, passive, and psychosocial modalities suggests that a setting-agnostic shared decision-making approach could entail the individual patient choosing from the menu of items within each subcategory to develop a more personalized multimodal approach that the patient is willing to use.

In conclusion, our findings indicate that there are many nonpharmacologic treatments that patients in ED are willing to try and that more than 90% of patients in ED are open to these modalities for managing their musculoskeletal pain. This study is one of the first we are aware of to directly elicit the patient perspective from those seeking care in the ED, and further research is needed to determine which of the identified treatments would be most feasible and efficacious to deliver in any given health care system. Furthermore, the clinical and biopsychosocial factors associated with willingness to try different subcategories of nonpharmacologic treatment methods are congruent with the characteristics of the patients most likely to derive benefit from those modalities. These findings are encouraging because they align with recent practice guidelines to increase the use of nonpharmacologic pain management strategies from the ED. Future research should include validation of these variables as predictors of nonpharmacologic treatment use, including performing inference analysis on these potential predictors, as well as determining the effect on clinical outcomes of nonpharmacologic interventions delivered from the ED matched with treatments that patients are most willing to try.

## Disclosures

This work was supported through internal funding by the Duke School of Medicine, including a Duke Faculty Flex Voucher and departmental support by the Division of Emergency Medicine, Department of Surgery and the Department of Orthopaedic Surgery. The authors have no conflicts of interest to declare.

## Appendix A. Supplemental digital content

Supplemental digital content associated with this article can be found online at http://links.lww.com/PR9/A165.
